# The relationship between *C. psittaci* infection and respiratory failure based on respiratory microbiota analysis: a retrospective cohort study

**DOI:** 10.3389/fmed.2026.1899239

**Published:** 2026-08-20

**Authors:** Lulu Chen, Ling Ye, Xianming Li, Haiting Zhou

**Affiliations:** Department of Respiratory and Critical Care Medicine, People's Hospital Affiliated With Fujian University of Traditional Chinese Medicine, Fuzhou, China

**Keywords:** *C. psittaci*, clinical characteristics, microbiome, next-generation sequencing, respiratory failure

## Abstract

**Objectives:**

This study aimed to explore the impact of the pulmonary microbiota on the occurrence of respiratory failure in patients with psittacosis pneumonia.

**Methods:**

A total of 20 patients diagnosed with psittacosis pneumonia were enrolled in this study. Patients were divided into two groups according to the presence or absence of respiratory failure at admission. Clinical data, laboratory findings, and bronchoalveolar lavage fluid analyses were collected. Next-generation sequencing was employed to analyze the pulmonary microbiota.

**Results:**

In this cohort, patients with respiratory failure exhibited a significantly higher relative abundance sequences of ***C. psittaci*** compared with those without respiratory failure. Notably, Spearman correlation analysis indicated a significant negative correlation between ***C. psittaci*** relative abundance of sequences and CD4+ T-lymphocyte counts (*r* = −0.870, *p* = 0.002) and a positive correlation with the pulmonary arteriovenous oxygen partial pressure difference (*r* = 0.728, *p* = 0.017). Further analysis of the bacterial communities showed a negative association between *Pseudomonas* and ***C*.** ***psittaci*** (*r* = −0.509, *p* = 0.031), whereas *Veillonella* was positively correlated with ***C. psittaci*** (*r* = 0.533, *p* = 0.023).

**Conclusions:**

The relative abundance sequences of ***C. psittaci*** was significantly higher in patients with respiratory failure than in those without respiratory failure, suggesting an association between ***C. psittaci*** load and disease severity. The significant negative correlation between relative abundance sequences of ***C. psittaci*** and CD4+ T-lymphocyte count suggests that ***C. psittaci*** may exacerbate disease progression by compromising the host immune response, but direction of this relationship cannot be determined. Furthermore, the negative correlation between the relative abundance sequences of *Pseudomonas* and *C. psittaci* suggests a possible competitive or protective interaction between these two organisms. In contrast, the positive correlation between ***Veillonella*** and ***C*.** ***psittaci*** may indicate a shared ecological niche. These findings provide preliminary evidence of a relationship between lung microbiota composition and the severity of psittacosis pneumonia, while the causal mechanisms remain to be elucidated in future studies.

## Introduction

***Chlamydia psittaci*** (***C. psittaci***) is a Gram-negative obligate intracellular bacterium that primarily infects birds but can also affect mammals. This pathogen is transmitted to humans via the respiratory route and causes an acute disease known as psittacosis. The first recognized cases of human psittacosis occurred in the late nineteenth century, and major epidemics were reported worldwide between 1890 and 1930 (1). In humans, psittacosis typically presents with non-specific flu-like symptoms or as community-acquired pneumonia (CAP), accounting for approximately 1.03% of all CAP cases (2). Currently, most reported cases of psittacosis are identified by physicians in respiratory medicine departments and intensive care units (3). Respiratory failure is a severe complication of ***C*.** ***psittaci*** infection, and most such cases have resulted in clinical deterioration and death (4). In the twentieth century, the mortality rate of psittacosis was as high as 50% (5).

Most psittacosis cases are associated with a history of avian exposure, and this condition must be distinguished from other avian-transmitted infections, such as H7N9, H5N1, and H5N6. The difficulty in differentiating these pathogens partly explains the relatively high rates of severe cases and mortality among patients with psittacosis. Advanced age (over 65 years), male sex (6), and abnormal levels of creatine kinase and brain natriuretic peptide (7) have been reported as risk factors for severe psittacosis. Miyairi et al. (8) found that disease severity results from the varying capacity of different strains to trigger macrophage activation. Upper respiratory tract colonization is a prerequisite for lower respiratory tract infection in many bacterial respiratory pathogens. The respiratory microbiota protects against this initial step in the pathogenesis of respiratory infection, a phenomenon termed “colonization resistance,” which may be important for maintaining respiratory health (9). Beyond these mechanisms, the respiratory microbiome is presumed to be related to airway structural development and local immunity (10). In this regard, studies evaluating the microbiome in patients with lower respiratory tract infections have provided evidence that microbiome composition is associated with the clinical course. For example, patients with ventilator-associated pneumonia presented with an increased bacterial load and reduced bacterial diversity in their tracheal aspirates (11).

Recent literature on psittacosis has primarily focused on the clinical characteristics of the disease, with limited in-depth analysis of the occurrence of respiratory failure in patients with psittacosis-associated pneumonia. Furthermore, the effects of changes in the lower respiratory tract microbiota on the clinical course of ***C. psittaci*** pneumonia remain poorly understood. Based on the above data we hypothesized that lower respiratory tract microbiota composition in psittacosis pneumonia patients is related to disease severity, and that changes in certain bacterial taxa can affect respiratory failure by altering host immune status. We tried to describe the relation between lung microbiota composition and disease severity, characterize composition and study lung microbiota influence respiratory failure in psittacosis pneumonia patients.

## Material and methods

### Patients and diagnostic criteria

This study enrolled 20 patients diagnosed with psittacosis at the People's Hospital Affiliated with Fujian University of Traditional Chinese Medicine between January 2021 and December 2024. Demographic data, clinical features, laboratory findings, bronchoalveolar lavage fluid (BALF) analyses, imaging data, diagnostic and treatment information, disease course, and outcomes were obtained from the electronic medical records. Patients were divided into two groups according to the presence or absence of respiratory failure at admission: psittacosis pneumonia with respiratory failure and psittacosis pneumonia without respiratory failure. Pathogen diagnosis was established by clinicians based on imaging findings, clinical features, and laboratory test results.

The diagnostic criteria for psittacosis pneumonia were defined as follows: (I) fulfillment of the diagnostic criteria for CAP according to the Chinese adult guidelines for the diagnosis and treatment of CAP; (II) detection of specific ***C. psittaci*** gene fragments in next-generation sequencing (NGS) samples; and (III) findings consistent with the clinicians' diagnosis.

Respiratory failure was defined as a condition in which the arterial partial pressure of oxygen (PaO_2_) was less than 8 kPa (60 mmHg), with or without an arterial partial pressure of carbon dioxide greater than 6.65 kPa (50 mmHg), at sea level in the resting state while breathing room air. This definition excluded intracardiac anatomical shunts or intrinsic abnormalities in cardiac output.

### Collection and DNA extraction from clinical samples

The collection time of BALF was before the start of treatment after admission. Peripheral blood samples were drawn at the same time of BALF collection. BALF and peripheral blood samples were obtained, and written informed consent was provided by the patients or their legal representatives. BALF samples were obtained by experienced bronchoscopists using approximately 30 mL of sterile 0.9% saline, yielding approximately 10 mL of BALF per participant. To control for contamination introduced during preprocessing and downstream library construction, NC-4[Negative control (Jurkat-T cultured cells at 10^4^ cells/mL)] was used as a low-input full-process negative control rather than a nuclease-free water blank. NC-4 consisted of Jurkat-T cultured cells at 10^4^ cells/mL and was processed through the complete workflow, including sample pretreatment, nucleic acid extraction, and library preparation. After liquefaction, BALF samples were homogenized in a bead-beating tube for cell wall disruption and then centrifuged at 12,000 rpm for 3 min to remove cellular debris. Subsequently, 1 mL of the homogenized solution was aspirated, and nucleic acid was extracted using a magnetic bead-based nucleic acid extraction kit in accordance with broad-spectrum or targeted enrichment technology to enhance the recovery of pathogenic nucleic acids. This matrix-matched full-process control was used to evaluate background signals introduced during pretreatment, extraction, and library preparation under a low starting-biomass condition. A Positive control genomic DNA (PC-gDNA) positive control was also processed in parallel with non-blood specimens; sequencing runs were accepted PC-gDNA only when the expected positive-control organism, Brevibacillus sp., was detected at rpm >10.

A forward broad-spectrum enrichment method was applied to enhance recovery of pathogen-derived nucleic acids. This enrichment step was performed using the Q-mNGS 3.0 broad-spectrum targeted next-generation sequencing (bs-tNGS) strategy, which enriches pathogen-derived nucleic acids by multiplex PCR before sequencing. The primer design was intended to broadly cover more than 1,000 respiratory pathogens by targeting species-specific sequences for DNA and RNA viruses and conserved marker genes or regions for bacteria and fungi, including 16S rRNA, topA, rpoB, valS, and internal transcribed spacer (ITS) regions. Because this targeted enrichment strategy preferentially amplifies predefined pathogen-associated targets, the results were interpreted for pathogen detection and etiological assessment rather than as a quantitative representation of the native BALF microbiota composition. Library preparation was performed using the NGS Automatic Library Preparation System (MatriDx Biotech Corp., Hangzhou, China). DNA quality was assessed using a BioAnalyzer 2100 (Agilent Technologies, Santa Clara, CA, United States) and quantified by quantitative real-time polymerase chain reaction for adapter measurement prior to sequencing. The libraries were subsequently normalized to target 1 million (M) reads, multiplexed, and sequenced using a single-end 50 base pair (bp) sequencing strategy. For contamination control, irrelevant cell line–based control samples were processed in parallel throughout the procedure. The NC-4 control, consisting of Jurkat-T cultured cells at 10^4^ cells/mL, was processed in parallel throughout the entire procedure as the unrelated cell-line control.

### Next-generation sequencing (NGS)

All samples yielded between 0.05 and 1 million reads. Raw sequencing reads were initially subjected to quality filtering to remove short reads (length < 35 bp), low-quality reads, low-complexity reads, and adapter sequences. Host sequences were removed by alignment to the human reference genome (GRCh38.p13) from the National Center for Biotechnology Information (NCBI) using Bowtie2 (version 2.3.5.1; Johns Hopkins University, Baltimore, MD, USA). Clean reads were subsequently aligned against a custom in-house microbial database, which compiled sequences from the NCBI GenBank nucleotide (nt) and assembly databases, as well as sequences assembled from our pure fungal cultures, using Kraken2 (version 2.1.2; Johns Hopkins University, Baltimore, MD, USA; confidence threshold = 0.5) for rapid taxonomic classification. The aligned microbial reads were further validated through a second alignment against the microbial database using Bowtie2. When discrepancies were detected between the Kraken2 and Bowtie2 outputs, reads were classified using BLAST [version 2.9.0; National Center for Biotechnology Information (NCBI), Bethesda, MD, USA]. Microbial reads in a library were considered valid when the following criteria were met: sequencing data passed the Accuri C6 quality control thresholds (library concentration >50 pM, Q20 > 85%, Q30 > 80%), and the species was not detected in the negative control (NC) from the same run, or the reads per million ratio (sample/NC) was greater than or equal to 5. This threshold was established by the authors to distinguish true-positive results from background contamination. In addition to the sample-to-control RPM threshold, batch-level environmental signals were filtered during interpretation. Microorganisms detected across the entire batch with comparable normalized RPM levels were regarded as likely environmental contaminants and were excluded from positive reporting unless supported by a clearly higher sample-specific signal and clinical context.

The multiplex panel of this bs-tNGS platform contains different primer pairs targeting different marker genes (e.g., 16S rRNA, topA, rpoB, valS and ITS region for bacteria and fungi, species-specific sequences for viruses). No internal calibration or normalization was applied to correct for differences in amplification efficiency between targets, since the platform was designed for qualitative or semi-quantitative pathogen detection rather than cross-taxa ecological quantification. Raw read counts for different primer sets are not directly comparable across taxa, and the reported “relative abundance” values should be treated as sequence proportions of each target-specific amplification context, rather than true ecological abundance ratios.

### Statistical analysis

Statistical analyses were performed using SPSS version 19.0 (SPSS Inc., Chicago, IL, USA). Continuous variables were first assessed for normality using the Shapiro–Wilk test, given the small sample size (*n* < 50). Variables that met the normality assumption are presented as mean ± standard deviation (SD) and were compared between groups using the independent samples *t*-test. Variables that deviated from normality—including interleukin-6 (IL-6), CD4+ and CD8+ T-lymphocyte counts, procalcitonin (PCT), and the sequencing-derived relative abundance of *C. psittaci*—are presented as median (interquartile range, IQR) and were compared using the Mann–Whitney *U* test. Intergroup differences in continuous variables were compared using Student's *t*-test or the Wilcoxon rank-sum test, as appropriate, whereas categorical variables were analyzed using the chi-square test or Fisher's exact test. Associations between variables were evaluated using Spearman's or Pearson's correlation coefficients, as appropriate. Parametric data were expressed as the mean ± standard error, and non-parametric data were expressed as the median and interquartile range. Graphs were generated using R (version 4.2.1; R Foundation for Statistical Computing, Vienna, Austria). More specifically, unsupervised clustering and differential expression analyses were performed using core functions of the limma package, including voom, lmFit, and eBayes. Differences between groups were assessed using the plotMDS function in the limma package, and results were extracted using the topTable function and ordered according to the *p* value. Microbial read RPKM values were log2-transformed, and relative read abundance was analyzed. Comparisons of microbial composition and abundance among groups were performed using the limma package. A *p* value < 0.05 was considered statistically significant.

## Result

### Demographics and clinical characteristics

From January 2021 to December 2024, 20 cases of psittacosis pneumonia were enrolled, One case was excluded because of missing clinical information, resulting in 19 included patients. The mean age of the cohort was 66.2 ± 8.8 years (range, 51–81 years), and 11 patients (57.9%) were male. Clinical data analysis showed that 11 patients had type I respiratory failure based on arterial blood gas analysis, whereas nine patients did not have respiratory failure. Among the 19 patients, 3 (15.8%) had documented previous bird exposure, and 7 (36.8%) were identified as smokers, of whom four were current smokers. In addition, eight patients (42.1%) had prior medical histories, including chronic respiratory diseases (two cases), diabetes mellitus (two cases), heart disease (one case), and gastric cancer (one case). The duration of illness ranged from 2 to 20 days (mean 9.3 ± 5.1 days), and fever lasted for a mean of 3.6 ± 1.6 days. At admission, most patients had low blood oxygen saturation. The mean blood oxygen saturation was 93.3% ± 8.8%, the mean alveolar–arterial oxygen (P_A_−_a_O_2_) gradient was 89.4 ± 70.9 mmHg, and the mean oxygenation index was 276.5 ± 87.7. No significant differences in baseline demographic characteristics, comorbidity profiles, or smoking history were observed between groups. Detailed results are presented in Table 1.

**Table 1:** Demographic, clinical characteristics, laboratory indexes and clinical symptoms of patients with psittacosis pneumonia.

Group	Psittacosis pneumonia with respiratory failure	Psittacosis pneumonia without respiratory failure	*P-*value
Patient characteristics			
Age (years)	66.0 ± 7.9	66.4 ± 10.4	0.915
Male gender, *n* (%)	54.5% (6/11)	55.6% (5/9)	1.000
Smoking, *n* (%)	27.3% (3/11)	50.0% (4/8)	0.377
Underlying diseases, *n* (%)			
Basic diseases	63.6% (7/11)	12.5% (1/8)	0.059
Lung underlying diseases	9.1% (1/11)	12.5% (1/8)	1.000
Coronary heart disease	9.1% (1/11)	0.0% (0/8)	1.000
Tumor	9.1% (1/11)	0.0% (0/8)	1.000
Diabetes	18.2% (2/11)	0.0% (0/8)	0.485
Symptom pre-admission			
Length of fever in hospital (days)	8.8 ± 3.4	10.0 ± 6.9	0.631
Length of fever reduction (days)	4.0 ± 1.4	3.0 ± 1.6	0.139
Blood gas analysis at admission			
Partial pressure of oxygen (mmHg)	65.3 ± 11.2	80.1 ± 13.1	0.024
Oxygenation index	221.0 ± 52.3	355.8 ± 62.6	0.000
Difference of alveoli-arterial oxygen pressure (mmHg)	118.4 ± 79.6	47.8 ± 21.8	0.039
Laboratory indexes at admission			
W.B.C count (10^9^/L)	6.0 ± 2.6	6.2 ± 2.9	0.907
Lymphocyte count (10^9^/L)	0.6 ± 0.2	0.7 ± 0.2	0.519
Neutrophil-to-lymphocyte ratio	9.7 ± 5.4	8.3 ± 2.7	0.531
Platelet count (10^9^/L)	199.1 ± 69.3	256.6 ± 117.0	0.197
C-reactive protein (mg/L)	186.4 ± 72.5	160.0 ± 70.8	0.440
Blood CD4+T lymphocyte count	322.1 ± 299.4	462.0 ± 97.7	0.235
Blood CD8+T lymphocyte count	187.0 ± 269.7	279.3 ± 188.4	0.464
CD4+/CD8+	2.6 ± 1.3	2.8 ± 2.4	0.889
IL-6 (pg/mL)	234.4 ± 282.4	148.3 ± 68.1	0.629
LDH (U/L)	273.4 ± 101.7	276.1 ± 67.7	0.958
Procalcitonin (ng/mL)	0.4 ± 0.3	0.9 ± 1.7	0.411
PT (S)	13.9 ± 0.6	14.7 ± 0.5	0.043
APTT (s)	40.3 ± 8.9	39.3 ± 9.4	0.853
BUN (μmol/L)	4.2 ± 0.6	4.0 ± 2.3	0.805
Indicators of BALF (%)			
Relative abundance of *C. psittaci*	54.9 ± 28.5	12.3 ± 10.1	0.003
Percentage of lymphocyte	29.8 ± 19.4	32.1 ± 32.7	0.872
Percentage of neutrophils	29.0 ± 22.8	35.0 ± 31.0	0.683
Percentage of macrophages	40.7 ± 21.1	30.3 ± 15.1	0.327
Lymphocyte-to- macrophages ratio	1.1 ± 1.4	1.8 ± 3.0	0.564

APTT, activated partial thromboplastin time; BUN, blood urea nitrogen; LDH, lactate dehydrogenase; IL-6, interleukin-6.

### Laboratory characteristics

Laboratory findings revealed that most patients (89.4%, 17/19) had normal white blood cell (WBC) counts, whereas 78.9% (15/19) exhibited lymphopenia. C-reactive protein (CRP) levels greater than 100 mg/L were detected in 78.9% (15/19) of patients, and more than half had elevated lactate dehydrogenase levels. The ratio of lymphocytes to macrophages in BALF was 1.4 ± 2.2. No statistically significant differences in any of these laboratory parameters were observed between patients with and without respiratory failure. Detailed laboratory findings are summarized in Table 1.

### Analysis of pulmonary microbiota in *C. psittaci* infection

**Figure 1:**
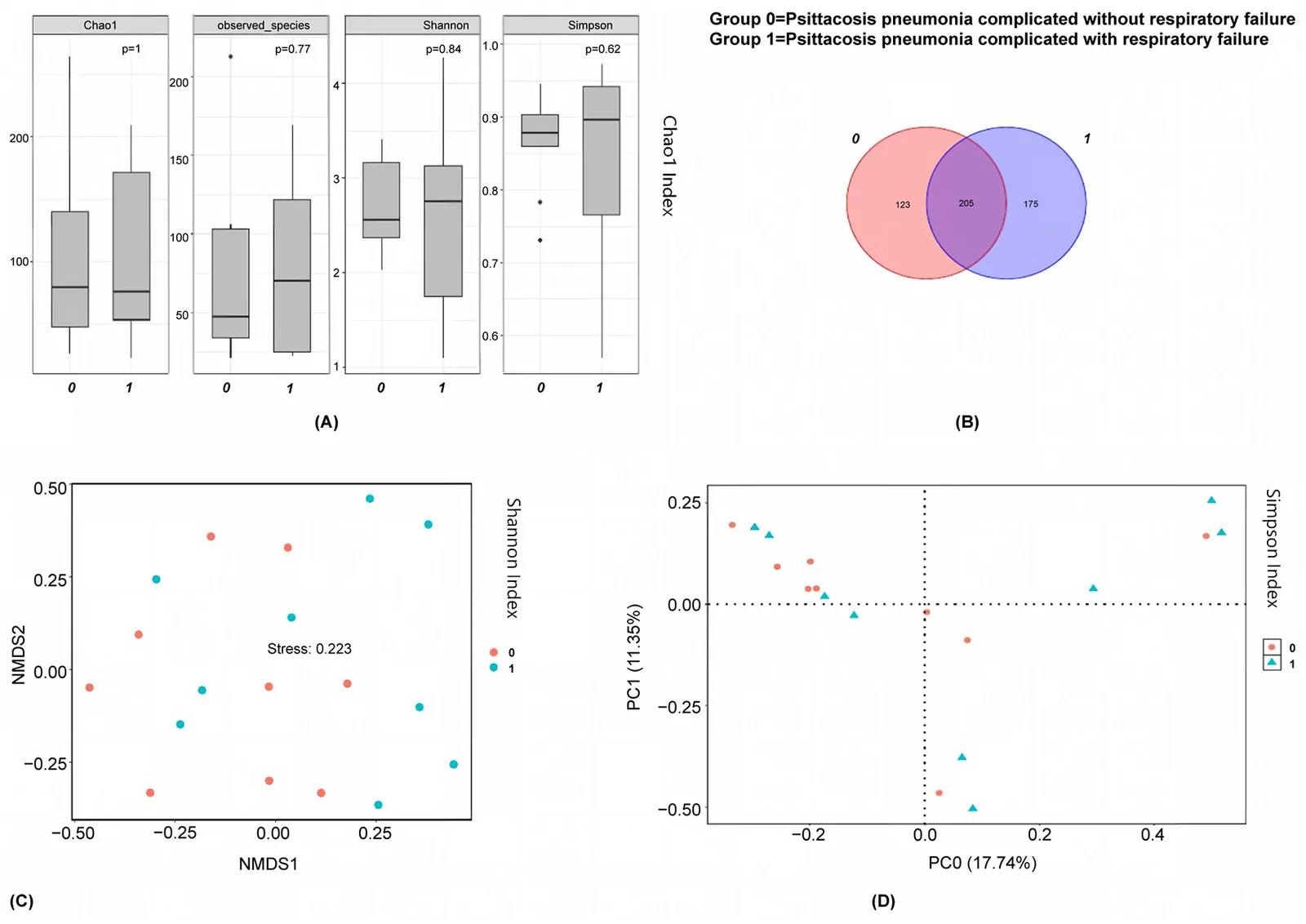
Alpha and beta diversity analysis of the pulmonary microbiota in BALF from patients with psittacosis pneumonia. **(A)** Comparison of alpha diversity indices—Chao1, observed species, Shannon, and Simpson—between Group 0 (without respiratory failure, *n* = 9) and Group 1 (with respiratory failure, *n* = 9). Boxplots display the median, interquartile range, and whiskers extending to 1.5 × the interquartile range. Between-group comparisons were performed using the Wilcoxon rank-sum test; no significant differences were observed for any index (all *p* > 0.05). **(B)** Venn diagram of the microbiome identified in BALF from patients with psittacosis pneumonia (total *n* = 18). The total number of shared microbial taxa between the two groups was 205, with 123 taxa unique to Group 1 (with respiratory failure) and 175 taxa unique to Group 0 (without respiratory failure). **(C)** Principal coordinate analysis (PCoA) based on Bray–Curtis distance. Each point represents an individual sample (Group 0, *n* = 9; Group 1, *n* = 9). Ellipses represent 95% confidence regions. Substantial inter-individual variability was observed within both groups, with no clear separation between groups. Formal statistical testing of beta diversity (e.g., PERMANOVA) was not performed due to limited sample size. **(D)** Non-metric multidimensional scaling (NMDS) analysis based on Bray–Curtis distance. Each point represents an individual sample. Similarly, considerable inter-individual variability and no distinct clustering between groups were observed.

**Figure 2:**
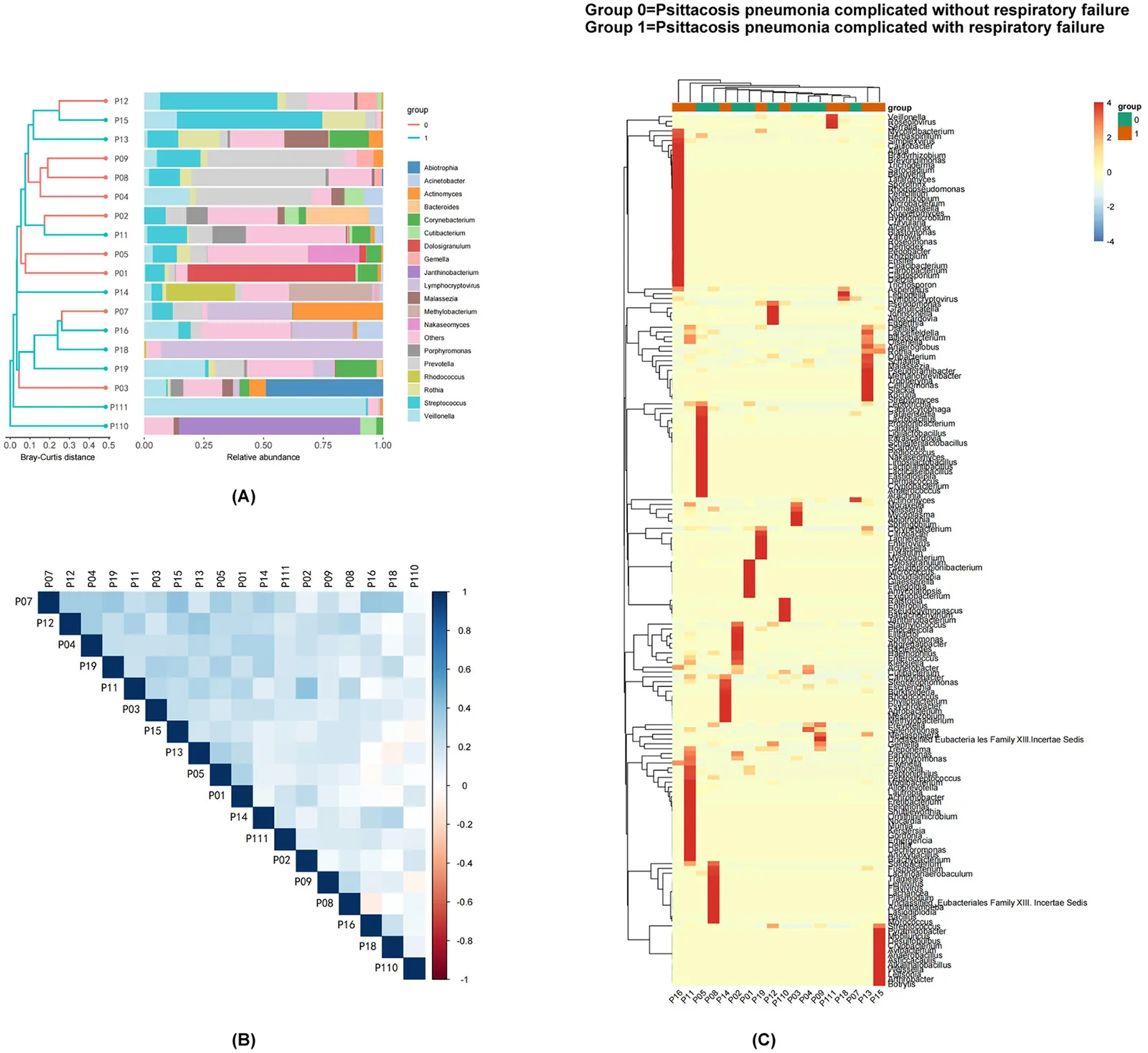
Microbial composition and inter-sample correlation analysis of BALF from patients with psittacosis pneumonia. **(A)** Bar plot of the relative abundance sequences of the top microbial genera identified in BALF. Taxa with mean relative abundance sequences < 1% across all samples were grouped as “Others.” The genera with relatively high overall abundance sequences were *Veillonella, Streptococcus*, and *Rothia*. **(B)** Inter-sample correlation heat map based on Spearman's rank correlation coefficient (*n* = 18 samples). Most correlation coefficients ranged between 0.2 and 0.4, indicating weak positive correlations between samples. No clear clustering of samples by group (Group 0, *n* = 9; Group 1, *n* = 9) was observed, consistent with high inter-individual variability in microbial community composition. **(C)** Relative abundance sequences heat map of the predominant microbial genera in BALF. Each column represents an individual patient sample, and each row represents a microbial genus. The color scale represents row *Z*-score–normalized relative abundance sequences. Each sample exhibited a distinct microbial profile, reflecting substantial inter-individual heterogeneity. The two patients diagnosed by BALF PCR rather than NGS were excluded from this analysis.

BALF samples from 18 patients infected with ***C. psittaci*** were analyzed by NGS, whereas the remaining two patients were diagnosed using bronchoalveolar lavage combined with polymerase chain reaction targeting *Chlamydia psittaci*. The results showed that patients with respiratory failure had a significantly higher relative relative abundance sequences of ***C*.** ***psittaci*** than those without respiratory failure. Both groups shared 205 species, with 123 species unique to the respiratory failure group and 175 unique to the non-respiratory failure group. Microbiota composition analysis indicated that ***Veillonella***, ***Streptococcus***, and ***Rothia*** were the most abundant genera. The relative abundance sequences heat map demonstrated distinct microbial compositions across samples, with marked interindividual variation. No statistically significant differences in alpha-diversity indices (Chao1, observed species, Shannon, and Simpson) were observed between the two groups. Principal coordinate analysis (PCoA) and non-metric multidimensional scaling (NMDS) based on community structure demonstrated substantial interindividual variability. To evaluate differences in overall microbial community structure between groups we performed Permutational Multivariate Analysis of variance (PERMANOVA) using Bray–Curtis distance and 999 Permutations. PERMANOVA result showed no difference in beta diversity between respiratory failure groups and non–respiratory failure groups (*R*^2^ = 0.062, *p* =0.357). Most samples, except for a few, were weakly correlated, with correlation coefficients primarily ranging from 0.2 to 0.4, indicating weak positive associations. Cluster analysis also showed no clear separation between the two groups. Interestingly, ***Stenotrophomonas*** and ***Burkholderia*** were detected in the respiratory failure group but not in the non-respiratory failure group. Detailed results are presented in Figures 1, 2. Diversity analysis demonstrated differences in pulmonary microbiota between patients with lung cancer and those with benign pulmonary nodules. Linear discriminant analysis effect size (LEfSe) was applied to further compare microbial communities and identify taxa with significantly different relative abundance of sequences between the groups, with the objective of identifying potential microbial biomarkers. The analysis identified five microbial genera with significantly different abundances at the genus level. Genera enriched in the respiratory failure group included *Stenotrophomonas* and *Burkholeria*.

### Pathogen–clinical index correlation in *C. psittaci* infection

**Figure 3:**
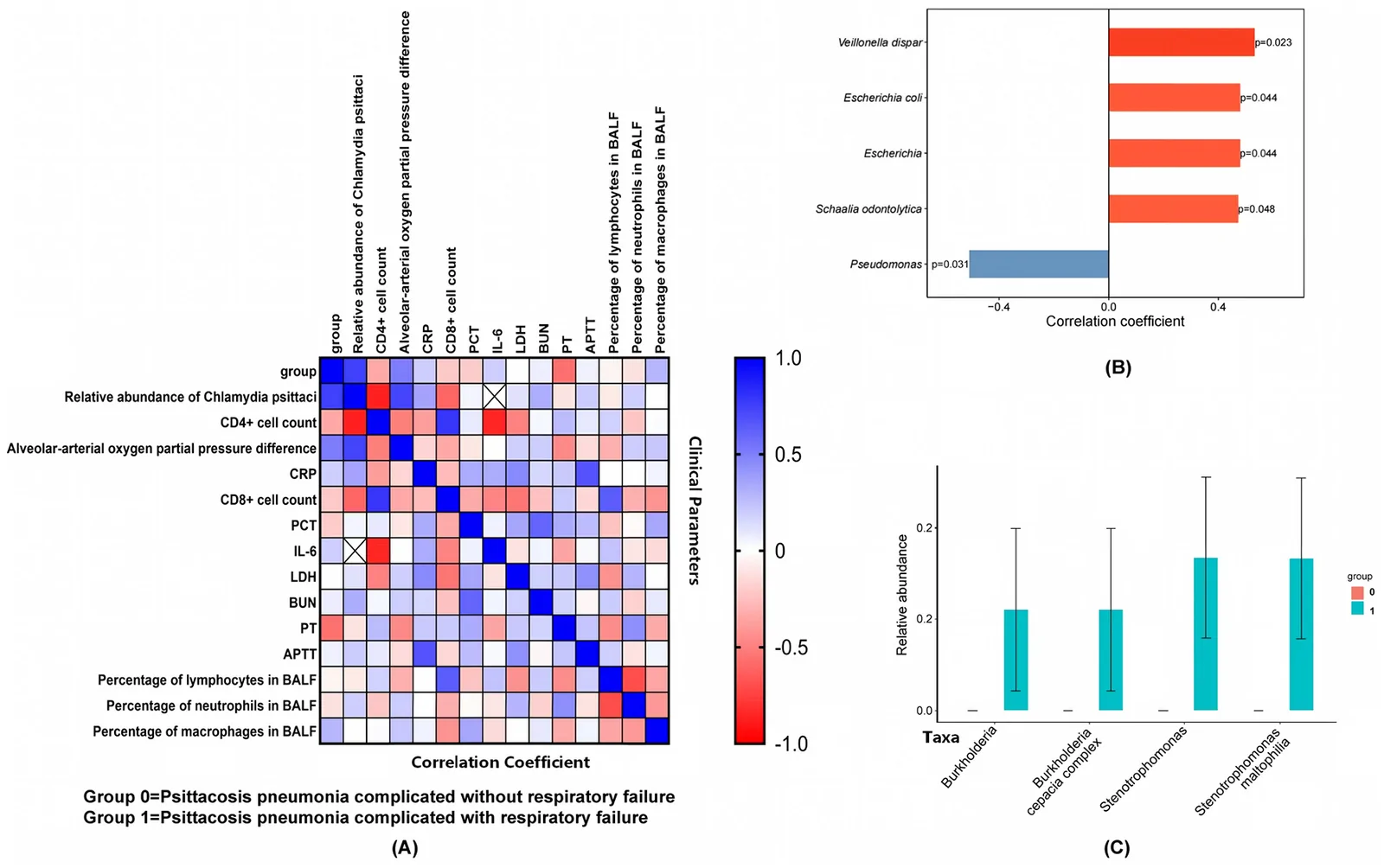
Correlation analyses between clinical indices, microbial taxa, and *C. psittaci* relative abundance sequences, and differentially detected taxa between groups. **(A)** Spearman correlation analysis examining the association between clinical indices and the relative abundance sequences of *C. psittaci* in BALF **(***n* = 18). Correlation coefficients (*r*) and corresponding *p* values are displayed. Relative abundance sequences of ***C. psittaci*** was negatively correlated with CD4+ T-lymphocyte count (*r* = −0.870, *p* = 0.002) and positively correlated with the alveolar–arterial oxygen partial pressure difference (PA–aO_2_; *r* = 0.728, *p* = 0.017). No significant associations were observed with CD8+ T-lymphocyte count, CRP, interleukin levels, or PCT (all *p* > 0.05). All *p* values are unadjusted for multiple comparisons. **(B)** Spearman correlation analysis between the relative abundance sequences of *Chlamydia psittaci* and other microbial genera in BALF (*n* = 18). The relative abundance sequences of ***Pseudomonas*** was negatively correlated with the relative abundance sequences of ***C. psittaci*** (*r* = −0.509, *p* = 0.031), whereas the relative abundance sequences of ***Veillonella*** was positively correlated with the relative abundance sequences of *C. psittaci* (*r* = 0.533, *p* = 0.023). All *p* values are unadjusted for multiple comparisons. These correlations are univariate in nature and were not adjusted for potential confounders; therefore, the reported associations should be interpreted as exploratory. **(C)** Microbial taxa detected exclusively or differentially between the two groups. ***Stenotrophomonas*** and ***Burkholderia*** were detected in Group 1 (with respiratory failure, *n* = 9) but not in Group 0 (without respiratory failure, *n* = 9). No formal differential relative abundance of sequences testing with multiple-testing correction was applied due to the limited sample size.

Spearman correlation analysis was performed to investigate the associations between clinical indices and the relative abundance sequences of ***C. psittaci*** in BALF. The relative abundance sequences of ***C. psittaci*** in BALF was negatively correlated with the peripheral blood CD4+ T-lymphocyte count (*r* = −0.870, *p* = 0.002) and positively correlated with the alveolar–arterial oxygen (PA–aO_2_) gradient (*r* = 0.728, *p* = 0.017). Additionally, the relative abundance sequences of ***C. psittaci*** was not significantly associated with CD8+ T-lymphocyte count, CRP, interleukin levels, or procalcitonin (PCT). Further analysis of the bacterial communities revealed ***Pseudomonas*** relative abundance sequences was negatively associated with ***C. psittaci*** relative abundance sequences (*r* = −0.509, *p* = 0.031), whereas ***Veillonella*** relative abundance was positively correlated with ***C. psittaci*** relative abundance sequences (*r* = 0.533, *p* = 0.023). These correlations are univariate and were not adjusted for confounders. The associations should be considered exploratory. Multivariate regression studies to determine independent association with respiratory failure were not feasible due to limited sample size, as detailed in the Limitations section. Detailed results are presented in Figure 3.

## Discussion

This study retrospectively analyzed the clinical features and pulmonary microbial spectra of patients with psittacosis pneumonia. ***C. psittaci***, belonging to the family Chlamydiaceae, is primarily transmitted to humans through contact with infected birds, their feces, secretions, aerosols, or dust (12). Epidemiological data indicate that this pathogen predominantly affects elderly patients with comorbidities. The study cohort included 20 patients with a mean age of 66.2 ± 8.8 years and an age range of 51–81 years. This age distribution is consistent with previous epidemiological reports. Psittacosis pneumonia has an insidious onset without typical early clinical manifestations and can rapidly progress from asymptomatic infection to severe pneumonia, potentially leading to respiratory failure and threatening patient safety.

Prior studies (13–15) have reported that patients with psittacosis pneumonia usually have normal WBC counts and decreased absolute lymphocyte counts. They also exhibit markedly higher CRP levels compared with those observed in bacterial pneumonia. These findings are consistent with our results. Unlike Yuan et al. (16), we observed no significant differences in lymphocyte count, serum CRP, or PCT levels between patients with and without respiratory failure. This discrepancy may be attributable to the limited sample size of our study. In particular, the small number of cases with respiratory failure may have reduced the statistical power. Additionally, all patients in our study had isolated psittacosis pneumonia without extrapulmonary organ involvement, unlike those in previously reported series. Furthermore, baseline characteristics, including fever duration, comorbidities, smoking history, and age, did not differ significantly between the two groups. The progression to severe pneumonia in psittacosis does not appear to be associated with baseline clinical characteristics or overall health status.

The relative abundance sequences of ***C. psittaci*** in BALF was significantly higher in patients with psittacosis pneumonia who developed respiratory failure than in those who did not, indicating a close association between relative abundance sequences of ***C. psittaci*** and disease severity. Additional analysis revealed a negative correlation between the relative abundance sequences of ***C. psittaci*** and peripheral blood CD4+ T-lymphocyte counts. As helper T cells, CD4^+^ T lymphocytes play a crucial role in the proliferation and differentiation of B cells, T cells, and other immune cells. Their function reflects overall immune status (17). Previous studies have shown that CD4+ T-lymphocyte counts are associated with disease progression and prognosis (16). Multivariate analyses (18–20) have demonstrated that a higher lymphocyte count was the only protective factor for survival in psittacosis pneumonia. From a pathogenic perspective, ***C. psittaci*** enters the lungs via the respiratory tract and is recognized by resident immune cells, thereby activating the pulmonary immune response and promoting the release of inflammatory mediators to combat the pathogen. This process leads to the recruitment of additional immune cells to the lungs, initiating cascades of inflammatory mediators and cytokines. If the pathogen is not eliminated promptly, inflammatory mediators further activate lymphocytes, thereby initiating an adaptive immune response. Persistent attempts to clear the pathogen may result in hyperactivation and apoptosis of lymphocytes, ultimately leading to immune dysregulation. An alternative explanation is equally plausible: a preexisting immunocompromised state, including reduced CD4+ T cell counts, may have impaired clearance of *Chlamydia psittaci*, permitting higher pathogen loads and worsened infection. Because this study used a retrospective observational design, the available data cannot distinguish between these possibilities, and both causal directions—infection causing immune exhaustion or immunodeficiency enabling increased pathogen burden—remain reasonable. Consequently, the causal relationship between relative abundance sequences of ***C. psittaci*** and reduced CD4+ T lymphocytes must be clarified by prospective studies and experimental evidence.

Microbial communities have co-evolved with humans for millions of years and inhabit all surfaces of the human body, including the respiratory mucosa (9). Diverse microbial communities occupy distinct ecological niches within the respiratory tract. Molecular and biochemical studies (21) have shown that these microflora contribute to both health and disease. In addition to participating in the structural maturation of the respiratory tract and local immune development (22), the respiratory microbiota secrete antimicrobial substances and may interfere with drug activity. They may also function as drug transporters or adjuvants and are, in some contexts, considered therapeutic agents themselves (21). The relationship between the lung microbiome and pneumonia is complex, multifactorial, and bidirectional (10). Disruption of the host–microbiome interplay may underlie inflammatory diseases. The absence of a normal microbiome or disruption of the lung microbial community results in an immature pulmonary immune system that cannot adequately defend against pathogens. Conversely, an abnormal lung microbiome may alter the immune microenvironment by secreting metabolites, triggering inflammation, and producing toxins. These toxins may modify host genomic stability and elevate levels of carcinogenic microbial metabolites, thereby contributing to disease development and progression (23). In the present study, we analyzed the microbiota in BALF from patients with psittacosis pneumonia and found no significant differences in bacterial richness, species evenness, or diversity between patients with and without respiratory failure. Yuan et al. (16) found that there was no statistically significant difference in lower respiratory tract microbiota diversity between *C. psittaci* pneumonia and severe *C. psittaci* pneumonia. An earlier study (11) reported that patients with pneumonia exhibited reduced diversity of the pulmonary microbiota, with the most affected genera including ***Pseudomonas***, ***Staphylococcus***, and ***Streptococcus***. The composition and diversity of the respiratory microbiome in patients with pneumonia have been observed to fluctuate during disease progression. Yang et al. (24) reported that lower airway microbiome α-diversity was reduced in patients with severe pneumonia, and more than half (53.8%) exhibited a microbial composition dominated by a potential pathogenic bacterium (>50% relative abundance sequences). At the same time, the microbial composition of sputum in severe cases changed significantly across consecutive time points, with differences that were even greater than those observed at baseline. The discrepancies between the results of the present study and previous studies may be attributable to the absence of psittacosis patients with respiratory failure severe enough to require mechanical ventilation in our cohort. Consequently, disease severity in our patients may not have progressed to a stage at which microbial community diversity was markedly reduced. However, it remains unclear whether the lung microbiome changes dynamically as the disease evolves. Additionally, it is uncertain whether alterations in microbial diversity can serve as early predictors of progressive lung disease. Therefore, further investigation and longitudinal clinical studies are required to validate these findings.

***Veillonella***, ***Streptococcus***, and ***Rothia*** were identified as relatively abundant genera in BALF from patients with psittacosis pneumonia. ***Veillonella*** and ***Streptococcus*** have been recognized as core components of the lung microbiota in healthy individuals (25). ***Rothia***, which belongs to the family Actinomycetaceae, predominantly colonizes the oral cavity and throat in humans and is a facultative anaerobe. It is one of the dominant microorganisms in the lungs of patients with chronic obstructive pulmonary disease, cystic fibrosis, and bronchiectasis. ***C. psittaci*** infection may alter the alveolar microbiome and is potentially associated with local immune changes in the lung secondary to infection. However, the causal relationship between lung dysbiosis and disease progression remains unclear. Rigauts et al. (26) demonstrated that the presence of ***Rothia*** in the airway may be beneficial. Specifically, ***Rothia mucilaginosa*** within the airway microbiome can alleviate airway inflammation by inhibiting the NF-κB pathway. This study identified statistical associations between ***C. psittaci*** and other pulmonary microbes. Specifically, ***pseudomonas*** relative abundance sequences correlated negatively with relative abundance sequences of ***C. psittaci***, while ***Veillonella*** relative abundance sequences correlated positively. These findings reflect data-driven associations rather than direct evidence of functional interactions. Previous studies have proposed explanatory frameworks for such patterns, but those hypotheses remain to be validated. Wootton et al. (27) utilized pyrosequencing to analyze sputum specimens from adults with CAP and found that ***Veillonella*** and ***Streptococcus*** were the most abundant taxa. Dickson et al. (28) reported that a ***Streptococcaceae***-dominant lower airway microbiome may promote pathogen colonization and replication through the production of virulence factors or enhanced nutrient availability in the alveoli. It has also been reported that ***Veillonella*** induces biofilm formation by ***Streptococcus*** in a species-specific manner, thereby protecting ***Streptococcus*** from elimination (29). Propionate produced by ***Veillonella*** metabolism may exert anti-inflammatory effects, potentially influenced by variations in the lung microenvironment and microbial composition. Against this background, we hypothesize that the positive correlation between *Veillonella* and ***C. psittaci*** indicates either a shared ecological niche or that *Veillonella* creates conditions favorable to the persistence of ***C. psittaci***. This hypothesis, however, requires validation in animal models or clinical studies. In contrast, a *Pseudomonas*-dominant microbiome may inhibit pathogen virulence and facilitate the reestablishment of a healthy lung microbiome (10). We observed a negative correlation between the relative abundances sequences of ***Pseudomonas*** and ***C. psittaci***. From this finding, we hypothesize that ***Pseudomonas*** may compete with ***C. psittaci***. The current data cannot determine whether ***Pseudomonas*** antagonizes ***C. psittaci*** virulence or mitigates the inflammatory response. Testing this hypothesis will require *in vitro* competition assays and *in vivo* animal-model experiments. An inverse correlation was observed between ***Pseudomonas*** relative abundance sequences and the relative abundance sequences of ***C*.** ***psittaci***. These findings indicate that perturbations in the lung microbiota may play a substantial role in promoting local and systemic inflammation. The development of psittacosis pneumonia results from complex interactions among the host immune system, the lower respiratory microbiome, and ***C. psittaci***. It has been hypothesized that dysregulation of the pulmonary microbiota may synergistically or additively influence the host immune response and pathogen virulence, thereby affecting both the pathogenesis of psittacosis pneumonia and the development of acute respiratory failure (30).

This study aimed to analyze the pathogenic spectrum and lung microbiota profiles in patients with psittacosis pneumonia using NGS. We also investigated the correlation between these profiles and clinical stratification. The relative abundance sequences of ***C. psittaci*** was negatively associated with PaO_2_, indicating a potential influence on pneumonia severity. In addition, we found that the relative abundance sequences of ***C. psittaci*** was negatively correlated with peripheral blood CD4+ T-cell counts and ***Veillonella*** relative abundance sequences, but positively correlated with ***Pseudomonas*** relative abundance sequences in BALF, indicating complex interactions among immune status, pathogenic bacteria, and the lung microbiome. These results suggest that complex interrelationships among immune status, pathogenic bacteria, and the lung microbiome. Evidence regarding the direct role of the local microbiome in the pathogenesis of psittacosis pneumonia remains limited. The microbiological co-abundance sequences patterns reported here produce testable hypotheses for future work. However, its influence on clinical manifestations and disease progression warrants further investigation. The findings of this study provide a foundation for subsequent research investigating the effects of ***C. psittaci*** infection on clinical outcomes and disease progression and contribute to a better understanding of the mechanisms underlying psittacosis pneumonia.

One of the important points of relative abundance data is that we used broad-spectrum targeted next generation sequencing (Q-mNGS 3.0) and PCR multiplex (PCR multiplex) with primer sets of different viral species and conserved marker genes (e.g., 16S rRNA, topA, rpoB, valS, ITS regions) of bacteria and fungi (see Materials and methods section). We selected this approach to maximize clinical sensitivity for detection of a wide range of respiratory pathogens such as fastidious and intracellular organisms such as *C. psittaci*. Since the amplitude efficiency differs greatly in primer–target pairs, read counts and “relative abundance” values do not represent true ratios between different bacteria in a sample. In one species and target genomes, relative abundance of *C. psittaci* sequences for patients can provide semi-quantitative proxy for pathogen load. Comparisons of relative abundance between genera (e.g., *C. psittaci*, vs. *Pseudomonas*, vs. *Veillonella*) are generally biased by differential amplification efficiency and therefore should not be taken as representing true ecological abundances. Platform-specific limitation differs from general compositional data issues related to relative abundance measures in standard 16S rRNA or shotgun sequencing (using universal primers or unbiased fragmentation) or statistical power constraints due to our small sample size. Cross-taxa correlations (e.g., *C. psittaci* and *Pseudomonas, C. psittaci* and *Veillonella*) should be taken as exploratory statistical associations describing co-detection patterns or amplification artifacts rather than ecological interactions. These observations produce hypotheses that need to be validated with uniformly applied amplification methods (shotgun metagenomics or quantitative PCR with standard absolute quantification).

### Limitations

Several limitations should be acknowledged in this study. First, the sample of patients with psittacosis pneumonia was relatively small (*n* = 20), and cases complicated by respiratory failure were especially scarce. This limited sample size reduced statistical power and prevented application of multiple-comparison corrections such as the False Discovery Rate (FDR), so some statistically significant findings may represent false positives. Likewise, because of the small sample, multivariate analyses could not be performed to assess independent associations between variables and respiratory failure, and potential confounders could not be adequately controlled. Similarly, the small sample size prevents multivariable analyses (e.g., logistic regression) to evaluate independent associations with respiratory failure and prevents enough control of confounders such as age, comorbidity, or previous antibiotic use. Thus, the associations are univariable and may be confounded and should be read cautiously. The small sample size also limited microbiome-specific analyses: PERMANOVA and LEfSe were performed but they were not statistically useful to detect small community differences or differentially abundant taxa. Microbial co-occurrence network analysis was not performed because reliable network inference relies on much larger sample sizes. In addition, all BALF samples were collected prior to in-hospital antibiotic administration to exclude the influence of inpatient antibiotics on respiratory microbiota, while complete pre-admission out-of-hospital antibiotic exposure information was unavailable from archived medical records due to retrospective data restriction, making it impossible to perform further stratified analyses to adjust for this key confounding factor, together with animal models, may help clarify these effects in future investigations. Lastly, the invasive nature of BALF sampling presents challenges for longitudinal monitoring of lung microbiome dynamics during disease progression and anti-chlamydial treatment. Alternative, less invasive approaches, such as sputum microbiome analysis or animal studies, may provide additional insight into the relationship between disease course and lung microbial communities. In summary, as a retrospective observational study, this work reveals only statistical associations due to methodological limitations. Future studies with larger sample sizes, prospective designs, and experimental validation are required to confirm these preliminary findings.

## Conclusion

In conclusion, we identified an association between pulmonary microbiota composition and the severity of psittacosis pneumonia. Patients with respiratory failure exhibited higher ***C*.** ***psittaci*** relative abundance of sequences, which was negatively correlated with peripheral blood CD4+ T-lymphocyte counts and positively correlated with the PA–aO_2_ gradient. Taken together, these findings may have important clinical implications. Moreover, we observed a negative correlation between ***Pseudomonas*** and ***C. psittaci*** and a positive correlation between ***Veillonella*** and ***C. psittaci***, suggesting a potential role for the pulmonary microbiota in disease progression. Because this study used a retrospective observational design, the relationships among these microorganisms should be interpreted as associations rather than causal mechanisms, and their precise interactions require confirmation by further experimental studies.

## Data Availability

The datasets presented in this study can be found in online repositories. The names of the repository/repositories and accession number(s) can be found below: https://ngdc.cncb.ac.cn/gsa/, PRJCA049978.
